# Effect of a homemade diet compared to a commercial diet on glycaemic variability and glycaemic control assessed by continuous glucose monitoring system in diabetic dogs: a randomised crossover study

**DOI:** 10.1111/jsap.70022

**Published:** 2025-08-22

**Authors:** A. M. Tardo, C. G. Vecchiato, E. Gherlinzoni, A. Corsini, S. Corradini, F. Del Baldo, G. Biagi, F. Fracassi

**Affiliations:** ^1^ Department of Veterinary Medical Sciences University of Bologna Ozzano dell’Emilia Italy; ^2^ Department of Veterinary Sciences University of Parma Parma Italy; ^3^ AniCura Clinica Veterinaria dell’Orologio Sasso Marconi Italy

## Abstract

**Objectives:**

To evaluate the effects of a homemade diet and a commercial diet on glycaemic control and glycaemic variability of diabetic dogs monitored with the FreeStyle Libre continuous glucose monitoring system.

**Materials and Methods:**

Prospective randomised crossover study including ten client‐owned diabetic dogs on insulin treatment with good glycaemic control. Dogs were randomly assigned to receive either a moderate‐fibre (total dietary fibre: 2.2 g/100 kcal ME) homemade diet or a high‐fibre (total dietary fibre: 4.8 g/100 kcal ME) dry commercial diet in a 2 × 6‐week period. Dogs were re‐evaluated every 2 weeks. Clinical and clinicopathological variables, selected continuous glucose monitoring system‐derived and glycaemic variability metrics, glucose nadir and postprandial hyperglycaemia were recorded. Differences between diets were analysed by a repeated measure ANOVA fitting a crossover design with pairwise comparisons.

**Results:**

There were no differences in insulin dose and glycaemic control levels between the two dietary periods. The homemade diet significantly reduced serum cholesterol concentration (mean difference: 76 mg/dL; 95% CI: −51.97 to 204 mg/dL). The percentage of time above glucose range was significantly lower (mean difference: −22.5%; 95% CI: −43.9% to −1.08%) and the percentage of time below range higher (mean difference: 6.9%; 95% CI: 1.38% to 12.4%) during the homemade diet period. The percentage of time in range and glycaemic variability metrics were not different between the two diets.

**Clinical Significance:**

The homemade diet and commercial diet can be considered valid dietary options in diabetic dogs. The results suggest that, with regard to the diets examined, the homemade diet might have a more effective glucose‐lowering effect compared to the commercial diet.

## INTRODUCTION

Diet plays a crucial role in the management of dogs with diabetes mellitus (DM) (Nelson, 2015). Several pet food companies offer commercial diets (CD) specifically formulated for diabetic dogs. While the composition of these diets varies, most are characterised by moderate to high fibre, high‐quality protein and restricted fat content (Nelson, 2015; Parker & Hill, 2023). Additionally, all dry diabetic CD and the majority of wet CD contain digestible ‘complex’ carbohydrates (CHO), primarily in the form of starch (Parker & Hill, 2023). The starch content in pet food can vary significantly, with higher levels typically found in dry products, where starch is essential for the formation of extruded dry kibble (Parker & Hill, 2023). Most studies assessing the role of nutrition in the glycaemic control of diabetic dogs have focused on the effects of dietary fibre and CHO (Blaxter et al., 1990; Elliott et al., 2012; Fleeman et al., 2009; Graham et al., 2002; Kimmel et al., 2000; Nelson et al., 1998). However, there is currently no consensus on recommended types and levels of dietary fibre and CHO in diabetic pet foods. The choice of diet ultimately depends on the weight of the diabetic dog, concurrent diseases and both owner and dog preferences (Nelson, 2015). Although the majority of pet owners prefer CD, some are interested in providing a homemade diet (HMD) for their animals (Oliveira et al., 2014). The preparation of HMD may enhance owners’ sense of involvement with their pets, and anecdotal evidence suggests that this practice is increasing (Remillard, 2008). HMDs could prove beneficial for dogs with DM, as their nutritional content can be customised to meet the individual patient’s needs, and clinical trials evaluating its use in client‐owned diabetic dogs are warranted.

The Freestyle Libre (FSL) continuous glucose monitoring system (CGMS) has revolutionised the management of DM in dogs (Corradini et al., 2016; Del Baldo, Canton, et al., 2020; Del Baldo & Fracassi, 2023; Shea & Hess, 2021). This device enables real‐time and comprehensive assessment of glucose trends and time spent within target ranges (Del Baldo & Fracassi, 2023), allowing clinicians to make faster and more informed decisions about insulin dose titration (Tardo et al., 2024). Moreover, thanks to CGMS, various metrics assessing glycaemic variability (GV), which refers to glycaemic excursions throughout the day (within‐day GV) or on different days (between‐day GV), are now affordable. In human medicine, GV is emerging as an additional glycaemic target due to its association with short‐ and long‐term diabetic complications (Ceriello et al., 2019; Jung, 2015). Additionally, in diabetic people, there is growing evidence that GV can be influenced by several nutritional factors, including types and levels of CHO, protein and fibre content of the diet (Breyton et al., 2020; Camps et al., 2021; Rasmussen et al., 2020; Tay et al., 2015; Tettamanzi et al., 2021). In veterinary medicine, the concept of GV has gained attention in recent years (Krämer et al., 2020; Linari et al., 2022; Miller et al., 2021; Zeugswetter & Sellner, 2020; Zini et al., 2018), but there are currently no studies that have evaluated the effects of nutritional factors on the GV of diabetic dogs.

The aim of this randomised crossover study was to evaluate the effects of an HMD and a dry CD on glycaemic control and GV of client‐owned dogs with stabilised DM, monitored with CGMS. The study was designed to minimise the influence of non‐dietary variables and facilitate a thorough evaluation of the effectiveness of nutritional therapy. The underlying hypothesis was that the HMD would provide glycaemic control comparable to that of the CD, but with greater GV, potentially due to increased variation in nutrient composition and ingredient preparation.

## MATERIALS AND METHODS

### Animals

Diabetic dogs receiving insulin treatment were recruited from 3 referral centres and prospectively enrolled in the study between September 2021 and December 2022. Diagnosis of DM was performed according to the Agreeing Language In Veterinary Endocrinology (ALIVE) criteria established by the European Society of Veterinary Endocrinology (ESVE, 2020). Dogs were eligible if they had been diagnosed with DM for at least 3 months, the type of insulin had not been changed in the 30 days preceding admission, and glycaemic control was deemed ‘stable’ (ALIVE Diabetic Clinical Score ≤ 3, Supporting Information S1) at the time of enrolment. Dogs not compliant with the dietary regimen, those with a relevant concurrent disease requiring a specific diet (e.g. renal therapeutic diet), and dogs that had received systemic or topical glucocorticoids or were diagnosed with diabetic ketoacidosis (DKA) within the previous 30 days were excluded. The trial was approved by the Scientific Ethics Committee of the University of Bologna (protocol number 296279/2021), and informed consent was obtained from each dog owner at the time of enrolment. The recruitment of dogs in the study was voluntary; the only cost for the owners was the purchase of ingredients listed in the HMD recipe, while the dry CD was provided at no cost.

### Diets and study design

The study was a prospective, randomised, crossover study. Using an online software program (Research Randomizer, Computer software, http://www.randomizer.org/), dogs were randomly assigned to one of the two diet periods: CD‐HMD or HMD‐CD. Each diet was fed for a 6‐week period, with a 5‐day transition in between, and the crossover design ensured that each dog received the two diets. The CD was a commercially available therapeutic veterinary diabetic dry diet for dogs (Monge VetSolution Diabetic, Monge & C. SpA, Monasterolo di Savigliano, CN, Italy), while HMD was a HMD formulated to be nutritionally complete and designed to be similar to CD in terms of protein, fat and starch content. The ingredients for HMD were chosen to closely resemble those used in the CD, while also taking into account their availability on the market, to ensure consistency and ease of replication by pet owners. Unlike the CD formulation, tapioca was excluded from the HMD due to limited availability, and the exclusive use of potatoes as the sole carbohydrate source was avoided to prevent large dietary volumes. Characteristics and chemical composition of the two experimental diets are shown in Table 1. The HMD was recreated in the laboratory and proximate analyses of the experimental diets (HMD and CD) were conducted according to International Standard methods (AOAC, 2000).

**Table 1:** Chemical compositions and ingredients of commercial diet (CD) and homemade diet (HMD) fed to diabetic dogs

	On DM basis (%)		g/100 kcal ME*	
	CD^†^	HMD^‡^	CD^†^	HMD^‡^
Crude protein	33	35	8.9	8.0
Crude fat	12	15	3.4	3.6
Starch	25	26	6.8	6.8
Ash	9	5	2.6	1.2
Crude fibre	6.6	2.4	1.8	0.6
Total dietary fibre	18	8.6	4.8	2.2
Soluble dietary fibre	2.9	2.0	0.8	0.5
Insoluble dietary fibre	14.8	6.6	4.0	1.5
Moisture (%)			5	73
ME (Kcal/100 g)*			348	118

CD Commercial Diet, DM Dry matter, HMD Homemade diet, ME Metabolisable energy

* ME calculated according to NRC 2006

^†^ Monge VetSolution Diabetic for dogs. Ingredients: dried chicken meat, tapioca (20%), potatoes (14%), pea fibre, dried fish (anchovy), dried eggs, salmon oil, dried duck meat, brewers’ yeast, minerals, chicken oil, Xylo‐Oligosaccharides (XOS 0.4%), fenugreek saeed (0.15%), products and by‐products from processing fresh fruits and vegetables (melon juice concentrate – Cucumis melo cantalupensis – source of superoxide dismutase 0.005%), milk protein powder. l‐carnitine (260 mg/kg)

^‡^ Ingredients: Fresh chicken breast (49%), pearled barley (20%), peas (16%), potatoes (8%), lard, vegetable oils (sunflower, wheat), minerals and vitamins supplement (Essential Cane Adulto, Chemivit), salmon oil (EPA+DHA 31%), calcium carbonate, l‐carnitine.

Comprehensive instructions on preparing the HMD recipe and determining the daily feeding amount were provided to the owners. The daily feeding amount for each diet was established based on each dog’s nutritional needs, ensuring no unintentional body weight changes during the study. To achieve this, the caloric intake from the previous diet was maintained and adapted to the experimental diets. For each diet, the daily amount was divided equally between two meals, regardless of the type of insulin the dog was receiving. At the time of inclusion, the insulin dosage was kept unchanged; any necessary adjustments were made at the first available re‐evaluation.

### Evaluations

Baseline data were collected at the time of inclusion in the study (T0) and re‐evaluations were performed every 2 weeks (T2‐T4‐T6 for each dietary period) thereafter. At T6, the dietary regimen was changed (e.g. from CD to HMD and vice versa). During each evaluation, the following were performed: recording of ALIVE Diabetic Clinical Score (ESVE, 2020) based on owner perception of clinical signs, body weight, body condition score (BCS), clinical hypoglycaemic events and unusual clinical signs (e.g. vomiting or diarrhoea) in the previous two weeks; assessment of IG data and application of a new Freestyle Libre® sensor; insulin dose adjustment.

Owner perception of clinical signs and assessment of IG data informed insulin dose adjustments and final categorisation into level of glycaemic control (maximum score 12: 0 to 3 good control, 4 to 8 moderate control, 9 to 12 inadequate control).

Blood and urine samples were collected at baseline (T0) and at the end of each 6‐week dietary period (T6). At the time of blood collection, the dogs had to be fasted for at least 12 hours. Measurement of serum fructosamine concentrations (Fructosamine 17350H, Sentinel Diagnostic, Milano, Italy) (Del Baldo, Magna, et al., 2020), Chemistry profile (AU 480, Beckman Coulter/Olympus, Brea, CA) and urinalyses were performed by standard laboratory methods at the internal laboratory of the Veterinary Teaching Hospital of the University of Bologna.

### Continuous glucose monitoring system

The IG measurements were acquired with a validated CGMS (FreeStyle Libre, Abbott Laboratories Ltd, Chicago, Illinois). In this study, sensor placement was performed as previously described (Corradini et al., 2016). More than one generation of FreeStyle Libre (i.e. Freestyle Libre 1 and Freestyle Libre 2) was used throughout the study. This device transfers IG data from the sensor to the FreeStyle LibreLink mobile application, and when the device is connected to the internet, the data is automatically uploaded to the LibreView system. LibreView is a free, secure, cloud‐based diabetes management system provided by Abbott, enabling remote data sharing with healthcare providers (Del Baldo & Fracassi, 2023). The system generates comprehensive glucose reports from the uploaded IG data, including the Ambulatory Glucose Profile (AGP). The AGP report provides both a visual and a statistical summary of the glucose metrics such as mean glucose (MG), percentage of time in range (TIR%), time above range (TAR%) and time below range (TBR%), along with GV expressed as percent coefficient of variation (CV%). At each time point, FSL‐derived metrics including MG, TIR% (70 to 250 mg/dL), TAR% (>250 mg/dL) and TBR% (<70 mg/dL) were recorded in a Microsoft Excel spreadsheet. The following GV metrics were computed by processing FSL data in a web‐based application (GlyCulator 3.0) (Chrzanowski et al., 2023): standard deviation of mean glucose concentration (SD), within‐day CV%, between‐day CV% and mean amplitude of glycaemic excursion (MAGE). The MAGE quantifies the average magnitude of glucose fluctuations that exceed one standard deviation from the 24‐hour mean glucose concentration and is considered the gold standard for assessing short‐term, within‐day GV in diabetic people (Vergès et al., 2022). Interstitial glucose concentrations were analysed for postprandial hyperglycaemia (PPH; 30, 60, 90 and 120 minutes after meal) and glucose nadir.

### Data analysis

Statistical analysis was performed using commercial statistical software packages (GraphPad Prism 9.5.1, San Diego, California). Descriptive statistics were generated to characterise the study population. The continuous data were assessed using the Shapiro–Wilks test for normality and reported as mean ± SD or median and range (minimum and maximum value), depending on whether the data were normally or not normally distributed, respectively. Mean differences and their 95% CI were computed. Categorical variables were described with frequencies, proportions or percentages.

Differences between variables were tested by a generalised linear model (GLM) fitting a crossover design with diet (CD and HMD) and period (CD‐HMD or HMD‐CD) as fixed factors, and subjects as random factors; period and diet × period interaction were tested to exclude any carry‐over effect. Post‐hoc analysis within the framework of the GLM was conducted using pairwise tests.

Differences between the two experimental diets for the clinicopathological variables measured at the inclusion (T0) and at the end of each 6‐week dietary period (T6) were assessed using Wilcoxon Rank‐Sum test or paired *T*‐test; statistical significance was set at P < 0.05.

## RESULTS

### Study population

A total of 10 client‐owned diabetic dogs were included in the study. The majority were purebred dogs (6/10: Miniature poodle, Deutsch Drahthaar, Epagneul Breton, Jack Russell Terrier, Labrador retriever, Cavalier King Charles Spaniel). Six spayed females and 4 males, one of which was intact, were included. At the time of enrollment, the median age was 9 years (range, 2 to 14), the median body weight (BW) was 10 kg (5.2 to 36.5), and the median BCS was 5/9 (3/9 to 7/9). Prior to inclusion in the study, all dogs were fed veterinary therapeutic dry diets. Specifically, 8/10 dogs were on diabetic diets from different pet food manufacturers than the one used in this study. The remaining two dogs were fed a weight‐loss diet and a gastrointestinal diet, respectively.

Dogs were treated with insulin glargine 300 U/mL (6/10 Toujeo, Sanofi‐Aventis Deutschland GmbH) or porcine lente (4/10, Caninsulin, Intervet International B.V.). All dogs receiving lente insulin were managed with twice daily insulin injections, while dogs receiving insulin glargine 300 U/mL were managed with once (in 3/6 dogs) or twice (in 3/6 dogs) daily insulin injections. Insulin type was not changed during the study. Only one dog had a concurrent disease, myxomatous mitral valve disease (ACVIM stage B1). Concurrent medications were recorded in one dog, which was receiving bezafibrate (Bezalip, Aurobindo Pharma, Italy), and the treatment was continued throughout the study without any dosage changes.

### Clinical and clinicopathological outcomes

**FIG 1:**
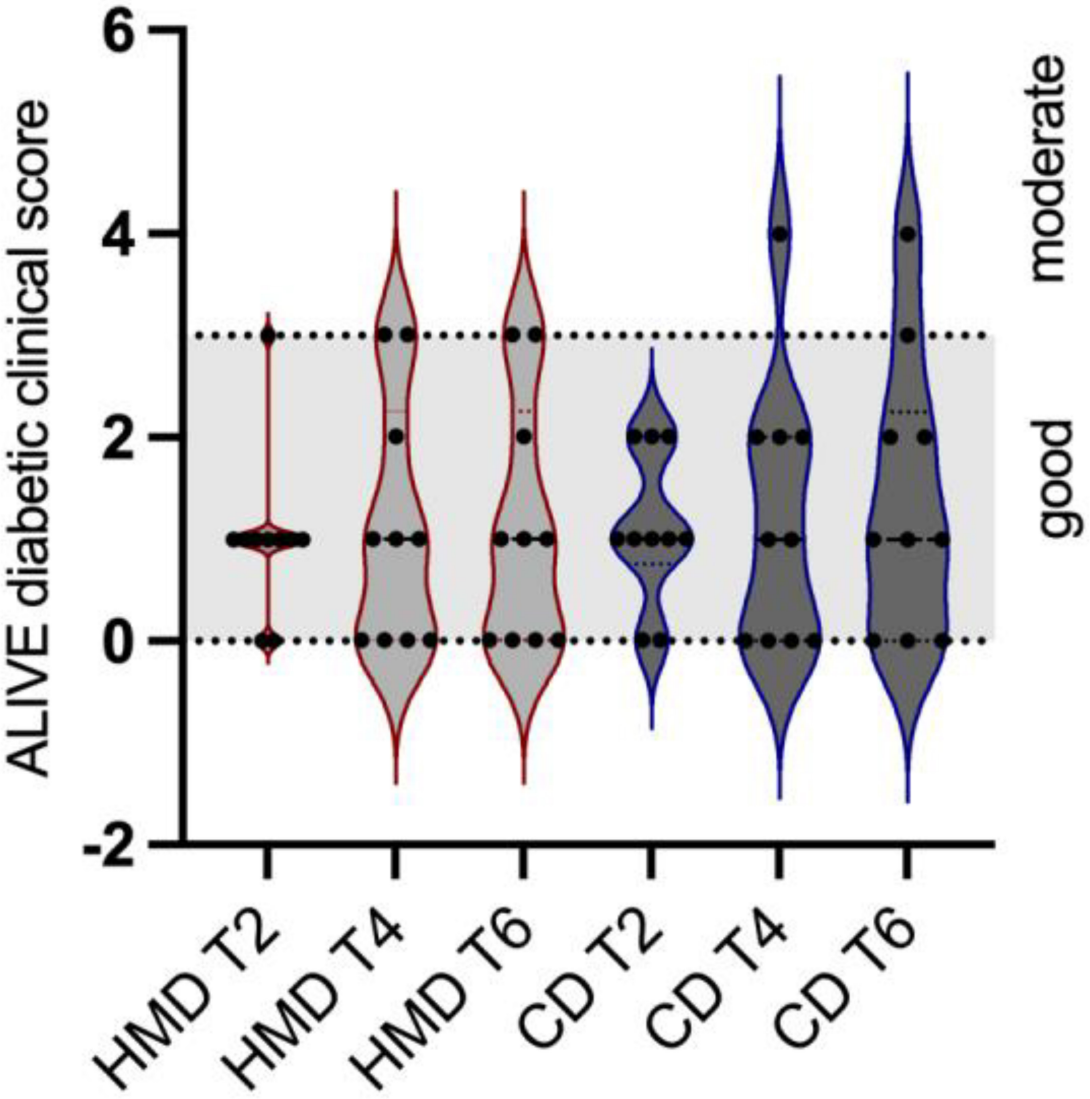
Violin plots showing the ALIVE diabetic clinical score recorded in 10 diabetic dogs (black dots) receiving both a commercial dry diet (CD) and a homemade diet (HMD) during periods of 6 weeks each, with follow‐up recorded every 2 weeks (T2, T4 and T6). The grey shaded area represents the range from 0 to ≤3, indicating good glycaemic control. In the violin plot, coloured dotted line represents quartile, black dotted line represents the median.

All dogs were compliant with the two dietary regimens, and none of the owners reported issues related to palatability of the diet or changes in faecal consistency. Body condition score did not significantly change throughout the study and between the two diets (P = 0.78). Median BW fluctuated between 10.13 kg to 10.7 kg (min: 5.1 to 5.45 kg; max: 34.9 to 36.5 kg), without significant changes (P = 0.69). As per inclusion criteria, all dogs enrolled had good diabetic control as assessed by the ALIVE score (median: 2, range: 0 to 3). When HMD was fed, 10/10 dogs had good glycaemic control at T6 (Median ALIVE score 3, range 0 to 3); while, when CD was fed, 9/10 dogs had good glycaemic control and 1/10 had moderate control (Median ALIVE score 3, range 0 to 4). However, differences between the two dietary periods were not significant (P = 0.87; Fig 1). Clinical hypoglycaemia was observed in one dog during the HMD period and in another during the CD period. Both episodes were mild in severity: one dog exhibited lethargy and weakness, while the other presented with lethargy, weakness and generalised tremors.

The median (range) insulin dose at the time of inclusion (baseline) was 1.1 (0.55 to 1.9) U/kg/day. While the median (range) insulin dose at T6 was 0.97 (0.54 to 2.53) U/kg/day in CD dogs and 0.95 (0.44 to 2.43) U/kg/day in HMD dogs. There were no differences in insulin dose between the two dietary periods (P = 0.32). For each dietary period, insulin dose adjustments were recorded across 30 evaluations (T2‐T4‐T6 for each of the 10 dogs). When HMD was fed, the insulin dose was increased in 10/30 (33%) evaluations, decreased in 6/30 (20%) and remained unchanged in 14/30 (47%). Conversely, when the CD was fed, the insulin dose was increased in 11/30 (37%) evaluations, decreased in 3/30 (10%) and remained unchanged in 16/30 (53%).

Clinicopathological variables assessed in diabetic dogs at baseline and at the end of each dietary period (T6) are shown in Table 2. Dogs receiving the CD had significantly higher serum cholesterol concentration at T6 compared to baseline (mean difference: 45.3 mg/dL; 95% CI: 0.33 to 90.3 mg/dL; P = 0.048). When fed HMD, dogs had a lower cholesterol level at T6 compared to CD (mean difference: −76.2 mg/dL; 95% CI: 18.36 to 134 mg/dL; P = 0.02), while other parameters were not different between the two diets.

**Table 2:** Clinicopathological variables measured in 10 diabetic dogs at the time of inclusion in the study (baseline) and at the end of each 6‐week dietary period (T6, CD or HMD)

Variable	Reference	Baseline	CD (T6)	HMD (T6)	P value
Fructosamine (μmol/L)	222 to 382	547 ± 56	549 ± 56	497 ± 93	0.07
Cholesterol (mg/dL)	123 to 345	337 ± 133*	382 ± 142*	306 ± 131	0.02
Triglycerides (mg/dL)	30 to 120	54 (43 to 583)	70 (43 to 269)	58 (29 to 321)	0.92
Creatinine (mg/dL)	0.75 to 1.40	0.83 ± 0.17	0.88 ± 0.12	0.88 ± 0.18	0.94
ALT (U/L)	15 to 65	69 (42 to 115)	69 (40 to 436)	80 (42 to 219)	0.63
β‐HBA (mmol/L)	0 to 0.9	0.04 (0.02 to 0.3)	0.03 (0.01 to 0.1)	0.03 (0.01 to 0.07)	0.63
Total protein (g/dL)	5.60 to 7.30	6.31 ± 0.36	6.41 ± 0.52	6.3 ± 0.44	0.39
Albumin (g/dL)	2.75 to 3.85	3.08 ± 0.26	3.15 ± 0.33	3.10 ± 0.3	0.13
USG	<1030	1049 (1035‐1060)	1050 (1032‐1064)	1040 (1020‐1054)	0.19
UPC	0 to 0.5	0.14 (0.09 to 0.24)	0.13 (0.09 to 0.45)	0.14 (0.07 to 0.31)	0.31
Glycosuria (mg/dL)	Absent	1000 (0 to 1000)	1000 (0 to 1000)	1000 (0 to 1000)	>0.99
Ketonuria (mg/dL)	Absent	2.5 (0 to 15)	2.5 (0 to 15)	0	0.25

Data are expressed as mean ± SD or median (range). The asterisk indicates statistically significant differences between baseline and T6 measured by *T*‐test. The P values indicate the results of *T*‐test between CD and HMD at T6

ALT Alanine aminotransferase, UPC Urine protein: creatinine ratio, USG Urinary specific gravity, β‐HBA β‐hydroxybutyrate acid

### Freestyle libre data analysis

**FIG 2:**
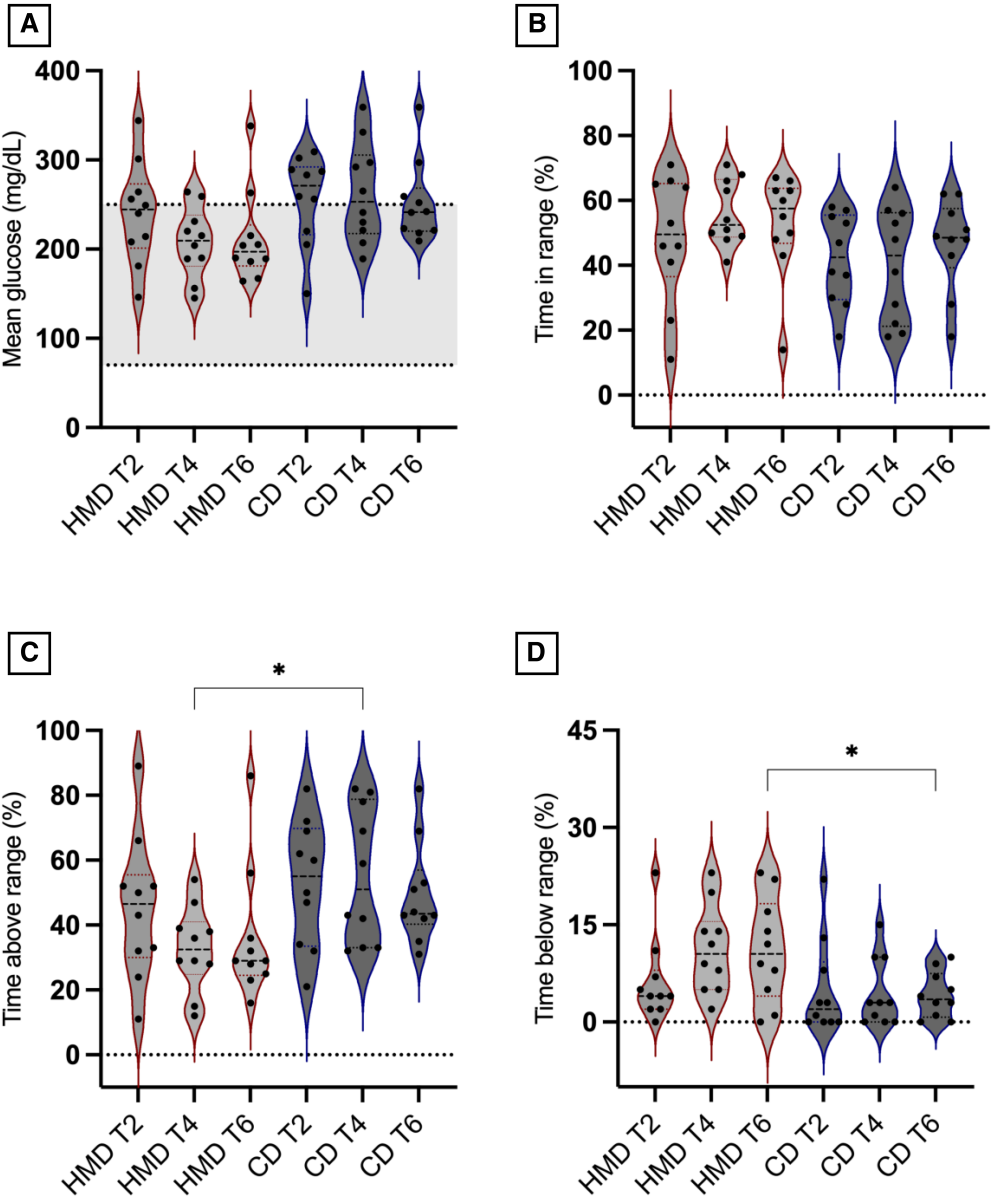
Violin plots showing: (A) the mean glucose; (B) the time in range (TIR %); (C) time above range (TAR%); (D) time below range (TBR%), in 10 diabetic dogs (black dots) receiving both a commercial diet (CD) and a homemade diet (HMD) during periods of 6 weeks each, with follow‐up recorded every 2 weeks (T2, T4 and T6). The asterisk indicates a significant difference (P < 0.05) by pairwise test between HMD and CD. The grey shaded area represents the glucose range between 70 and 250 mg/dL.

The median (range) sensor lifespan was 13 (4 to 14) days. A total of 59,219 IG concentrations were recorded. Freestyle Libre‐derived and GV metrics assessed in diabetic dogs during each dietary period (T2, T4 and T6) are shown in Table 3. The TAR% was significantly lower at T4 (mean difference: −22.5%; 95% CI: −43.9% to −1.08%; P = 0.04, Fig 2C) and TBR% higher at T6 (mean difference: 6.9%; 95% CI: 1.38% to 12.4%; P = 0.02, Fig 2D) during the HMD period; however, the MG (mg/dL, Fig 2A) and TIR% (Fig 2B) were not different between the two diets (P = 0.07 and P = 0.10, respectively). No differences in GV metrics were found between the two diets. The PPH 30 minutes after meal tended to be lower (P = 0.056) in dogs receiving HMD, while no differences were found for the other time points. Glucose nadir did not differ between HMD and CD (P = 0.31).

**Table 3:** Freestyle Libre‐derived and glycaemic variability metrics obtained in 10 diabetic dogs during each 6‐week dietary period (T2, T4 and T6)

Variable	Diet	T2	T4	T6	P value
Mean glucose (mg/dL)	HMD	240 ± 57	207 ± 39	212 ± 52	0.07
	CD	256 ± 50	263 ± 55	252 ± 45	
TIR%	HMD	48.6 ± 19.6	56.1 ± 10.1	52.9 ± 15.9	0.10
	CD	42.1 ± 14	40.3 ± 17.5	46.5 ± 14	
TAR%	HMD	45.2 ± 22.1	32.7 ± 13*	36 ± 20.5	0.04
	CD	52.9 ± 19.6	55.2 ± 21*	49.3 ± 15.5	
TBR%	HMD	6.2 ± 6.6	11.2 ± 6.7	11.1 ± 8*	0.03
	CD	5 ± 7.3	4.5 ± 5.3	4.2 ± 3.5*	
SD	HMD	86.5 ± 23	88.2 ± 23	85.3 ± 20	0.47
	CD	86.1 ± 16	74.4 ± 29	84.2 ± 15	
Within‐day CV%	HMD	43.8 ± 8.4	43.8 ± 8.4	43.6 ± 13.2	0.09
	CD	36.6 ± 10.8	34.5 ± 11.3	36.5 ± 9.5	
Between‐day CV%	HMD	47.3 ± 10	53.8 ± 8.3	51.9 ± 14.6	0.09
	CD	43.9 ± 13.9	43.2 ± 14.7	43.8 ± 10.1	
MAGE (mg/dL)	HMD	176 ± 53	184 ± 61	177 ± 58	0.83
	CD	173 ± 56	166 ± 51	167 ± 49	

Data are expressed as mean ± SD. The P value refers to the result of the generalised linear model, while the asterisk indicates the timepoint at which statistically significant differences between diets were observed by pairwise comparisons

CD Commercial diet, CV Coefficient of variation, HMD Homemade diet, MAGE Mean amplitude of glycaemic excursion, MG Mean glucose, SD Standard deviation of mean glucose concentration, TAR Time above range (>250 mg/dL), TBR Time below range (<70 mg/dL), TIR Time in range (70 to 250 mg/dL)

## DISCUSSION

Nutritional management plays a crucial role in the long‐term management of diabetic dogs. However, no consensus exists regarding the ideal composition and macronutrient balance in a dietary formulation for DM in dogs. The nutritional management of diabetic dogs has traditionally relied on commercially available diets, while alternative nutritional strategies, such as HMDs, have not been previously investigated in research studies. This gap in knowledge highlights the importance of investigating whether specific dietary interventions could improve management and potentially enhance the clinical outcome of canine diabetic patients. Considering the recent increase of HMDs in clinical settings, comparing this dietary approach with a standard therapeutic diabetic dry diet was considered of interest in the present study. In our study, diabetic dogs fed an HMD showed similar exogenous insulin requirements and glycaemic control levels as when fed a therapeutic veterinary diabetic dry CD. Therefore, our results suggest that the two dietary formulations used in this study are unlikely to result in clinically relevant differences when fed to individual diabetic dogs. Several hypotheses can be considered to explain our findings: (1) we included dogs with well‐controlled diabetes, carefully monitored, making it difficult to detect any worsening of clinical signs or significant changes in insulin dosage; (2) exogenous insulin likely has a predominant effect on glucose homoeostasis in diabetic dogs, potentially masking any subtle dietary influences on glycaemic control; or (3) the methods used to assess glycaemic control and insulin requirements may not have been sensitive enough to detect differences between the diets. For a more precise comparison between the two diets, we also evaluated metrics derived from the FreeStyle Libre®, which is currently the most widely used CGMS in veterinary medicine. In human medicine, an international panel of CGMS experts recently developed consensus guidelines to provide clinicians, researchers and individuals with DM with standardised recommendations for using, interpreting and reporting CGMS‐derived metrics in routine clinical care and research. These metrics are now regarded as supplementary glycaemic targets and outcome measures alongside glycated haemoglobin (Battelino et al., 2019, 2023). Although the FreeStyle Libre is increasingly used in diabetic dogs, its integration into routine clinical practice remains limited due to the absence of standardised guidelines for data interpretation. In our study, dogs receiving the HMD showed a reduced TAR% at T4 and an increased TBR% at T6 compared to those on the CD. These findings may suggest a more pronounced glucose‐lowering effect in dogs fed the HMD. This could be attributed to differences between the two diets, primarily in terms of ingredients and cooking processes. The starch sources varied between the diets (tapioca and potatoes in the dry diet vs. pearl barley and peas in the HMD), and it is known that starch plays a key role in modulating glucose fluctuations and lipidaemia in diabetic dogs (Teixeira et al., 2020; Teixeira & Brunetto, 2017).

In healthy dogs, studies have shown that diets with low carbohydrate content result in reduced postprandial glucose concentrations (Elliott et al., 2012; Hewson‐Hughes et al., 2011). Although the role of starch in the management of diabetic dogs has not been thoroughly addressed in studies so far (Teixeira et al., 2020; Teixeira & Brunetto, 2017; Teshima et al., 2021), managing carbohydrate intake is recommended to minimise postprandial hyperglycaemia (Behrend et al., 2018).

Sorghum‐ and barley‐based diets, as well as the inclusion of legumes such as lentils and peas, have been shown to induce better glycaemic responses in insulin‐treated dogs compared to rice‐ or corn‐based diets. These ingredients may contain components that help minimise postprandial hyperglycaemia in diabetic dogs, such as β‐glucan in barley (Teixeira & Brunetto, 2017; Teixeira et al., 2020), and the high resistant starch content in peas and lentils (Teshima et al., 2021). Barley and other cereals with a high content of dietary fibres or amylose have a low glycaemic index (Truswell, 1992); a concept from human medicine that has some applicability to dogs (Briens et al., 2021). The formulation of pet food is a more complex process than preparing a HMD because the extrusion process involves the interaction of different components besides their physical and chemical transformations. It is known that not only the type and source of starch, but also the different dietary components of the pet food have an impact on gelatinisation itself and the temperature at which gelatinisation occurs (Gibson & Alavi, 2013). Contrary to pet food production, preparing a HMD requires different ingredients to be cooked separately. The interactions between ingredients, for example starch‐lipid interactions or protein agglomeration, might impact the degree of starch gelatinisation in pet food but not in HMDs (Corsato et al., 2021). On the other hand, the cooking process of the HMD requires lower temperatures than the extrusion. This study did not standardise the cooking time of the HMD ingredients, as owners were simply instructed to cook barley and peas until tender, with barley having a slightly chewy texture. Moreover, after boiling, barley from the HMD may have preserved some resistant starch and reduced starch gelatinisation, potentially leading to decreased glucose absorption from the gastrointestinal tract (Teixeira & Brunetto, 2017). Additionally, the method of preserving barley potentially used by dog owners to avoid daily cooking (refrigeration at 4°C) may have influenced starch retrogradation. These hypotheses may also explain the tendency toward lower postprandial hyperglycaemia observed in the HMD group.

In our population of well‐controlled diabetic dogs, the mean TIR% was not different between the two diets and ranged from 40% to 56% when fed the CD and HMD, respectively. Notably, in people with type 1 and type 2 diabetes, it is recommended that over 70% of IG measurements fall within the target range (70 to 180 mg/dL) (Battelino et al., 2019). Our findings suggest that well‐controlled diabetic dogs may not meet the glycaemic targets recommended for diabetic people. However, establishing glycaemic target guidelines for diabetic dogs is beyond the scope of this study, and further research is warranted to assess this aspect.

In diabetic people, GV can be influenced by various nutritional factors, including the types and quantities of CHO, protein and dietary fibre (Breyton et al., 2020; Camps et al., 2021; Rasmussen et al., 2020; Tay et al., 2015; Tettamanzi et al., 2021). In this study, we analysed various metrics to assess GV throughout the day (SD, within‐day CV% and MAGE) and during different days (between‐day CV%). We found no significant differences between the two diets, which was unexpected given that the consistency and reproducibility of the diet are generally more challenging with HMD compared to CD. In a study evaluating pet owner perceptions regarding the use of HMDs, 30% of pet owners reported that they changed the diets, 40% did not adequately control the quantities of provided ingredients, and 56% indicated that their dog refused to eat at least one food item (Oliveira et al., 2014). In our study, we included highly motivated owners, and every re‐evaluation was attended by a nutritionist who verified the proper administration of all the ingredients of HMD and the daily amount of CD fed by the owners. The results of this study underscore the importance of considering owner commitment when using an HMD for the long‐term management of diabetic dogs.

Increasing the dietary fibre content is advantageous for managing overweight patients and is thought to enhance glycaemic control in diabetic dogs (Nelson, 2015). However, studies evaluating the effect of different fibre sources on diabetic dogs are still scarce. The diets used in this study markedly differed in the fibre content, with lower levels of total dietary fibre and insoluble fibre in the HMD, and to a lesser extent, soluble fibre. This can be attributed to the challenge of incorporating high amounts of insoluble fibre‐rich ingredients, which may negatively affect the palatability of HMD recipes. The ability of the soluble fraction of dietary fibre to form a viscous gel is critical, as it hinders the convective transfer of glucose and water to the intestinal absorptive surface, thereby slowing intestinal glucose absorption. Rapidly fermentable viscous soluble fibres (e.g. gums, pectin) impede glucose diffusion more effectively than insoluble fibres (e.g. cellulose, hemicellulose), making them more beneficial for glycaemic control (Nelson, 2015). In a previous study, diabetic dogs were fed, in a randomised model, dry diets differing in fibre and soluble fraction content. Dogs fed a diet high in fibre (73 g/Mcal) with low soluble fraction (<0.1 g/Mcal) had the best outcome in terms of glycaemic control (Kimmel et al., 2000). These findings were not confirmed by another study, in which wet diets differing in fibre content and source, as well as carbohydrate content, did not result in differences in terms of glycaemic control in diabetic dogs (Fleeman et al., 2009). Comparing these outcomes is challenging, as the diet highest in fibre in the study by Fleeman et al. (2009) is not comparable to that of Kimmel et al. (2000) in terms of total dietary and soluble fibre content, given that the former study used wet rather than dry diets. Therefore, the optimal fibre content and type for diabetic dogs has yet to be established, and individual factors such as body condition score and owner and dog preferences should also be considered. In this study, despite the difference in fibre types and content, body weight and BCS showed no significant changes when diabetic dogs were fed HMD or CD. This raises the question of whether high‐fibre diets should be routinely recommended in diabetic dogs.

In this study, when fed HMD compared to CD, dogs showed a reduction in serum cholesterol concentrations. The CD and the HMD had a similar fat content, with HMD being slightly higher compared to CD. Therefore, other factors than fat content might be involved in the cholesterol‐lowering effect exerted by HMD. A possible explanation can be found in the presence of barley in HMD, because similar results have been previously reported in diabetic dogs fed diets containing this cereal (Teixeira et al., 2020). Barley, in fact, contains ß‐glucans that interact with lipids and biliary salts in the bowel and consequently reduce cholesterol levels in humans (Sima et al., 2018). However, this mechanism cannot be confirmed in this study and in that of Teixeira et al. (2020) as the faecal bile acids excretion was not measured.

The present study has several limitations, including the small sample size. Additionally, only dogs with well‐controlled diabetes were included to minimise the influence of non‐dietary variables and allow for a thorough evaluation of the effectiveness of nutritional therapy. However, this selection criterion may have introduced bias into the results. In addition, glycaemic control was assessed using the ALIVE diabetic clinical score. While this tool offers a valuable standardised approach, it has certain limitations; for example, a dog may be classified as ‘stable’ despite exhibiting clinically significant signs of DM (e.g. a score of 3/12 in a dog with marked polyuria and polydipsia). Another limitation of the study is that in some dogs the CGMS was prematurely removed, which may have influenced the results of the FreeStyle Libre‐derived metrics. Furthermore, it was not feasible to ensure identical compositions between the two diets, particularly concerning fibre content. Finally, the diabetic dogs were managed at a referral centre, and the experimental conditions of this study may not be replicable in everyday veterinary settings.

In conclusion, both the CD and the HMD can be regarded as valid dietary options for managing DM in dogs. The HMD was associated with a significant reduction in the TAR% and cholesterol, and an increase in the TBR%. These results suggest that the HMD formulated for this study may have a more effective glucose‐lowering effect compared to the CD. In contrast to the initial hypothesis, GV metrics did not demonstrate significant differences when diabetic dogs were fed either the CD or the HMD. Further research is needed to confirm these findings and explore the long‐term effects of the HMD on glycaemic control and overall health in diabetic dogs. Future studies should aim to include larger sample sizes and diverse populations to enhance the generalisability of the results and ensure that these dietary options can be effectively integrated into clinical practice for optimal diabetes management in canine patients.

### Author contributions

**A. M. Tardo:** Conceptualization (equal); data curation (equal); formal analysis (equal); funding acquisition (equal); investigation (equal); methodology (equal); project administration (equal); resources (equal); visualization (equal); writing – original draft (equal). **C. G. Vecchiato:** Data curation (equal); formal analysis (equal); investigation (equal); methodology (equal); writing – original draft (equal). **E. Gherlinzoni:** Data curation (equal); investigation (equal); writing – original draft (supporting). **A. Corsini:** Investigation (equal); methodology (equal); writing – review and editing (equal). **S. Corradini:** Investigation (equal); methodology (equal); writing – review and editing (equal). **F. Del Baldo:** Supervision (equal); writing – review and editing (equal). **G. Biagi:** Supervision (equal); writing – review and editing (equal). **F. Fracassi:** Conceptualization (equal); funding acquisition (equal); methodology (equal); project administration (equal); supervision (equal); writing – review and editing (equal).

### Conflict of interest

No conflicts of interest have been declared.

## Supporting information


Data S1.

## Data Availability

The data that support the findings of this study are available from the corresponding author, upon reasonable request.

## References

[jsap70022-bib-0001] AOAC . (2000) Official methods of analysis of AOAC international, 17th edition. Gaithersburg, MD: Association of Analytical Communities.

[jsap70022-bib-0002] Battelino, T. , Alexander, C.M. , Amiel, S.A. , Arreaza‐Rubin, G. , Beck, R.W. , Bergenstal, R.M. et al. (2023) Continuous glucose monitoring and metrics for clinical trials: an international consensus statement. Lancet Diabetes and Endocrinology, 11, 42–57. 36493795 10.1016/S2213-8587(22)00319-9

[jsap70022-bib-0003] Battelino, T. , Danne, T. , Bergenstal, R.M. , Amiel, S.A. , Beck, R. , Biester, T. et al. (2019) Clinical targets for continuous glucose monitoring data interpretation: recommendations from the international consensus on time in range. Diabetes Care, 42, 1593–1603. 31177185 10.2337/dci19-0028 PMC6973648

[jsap70022-bib-0004] Behrend, E. , Holford, A. , Lathan, P. , Rucinsky, R. & Schulman, R. (2018) 2018 AAHA diabetes management guidelines for dogs and cats. Journal of the American Animal Hospital Association, 54, 1–21. 29314873 10.5326/JAAHA-MS-6822

[jsap70022-bib-0005] Blaxter, A.C. , Cripps, P.J. & Gruffydd‐Jones, T.J. (1990) Dietary fibre and postprandial hyperglycaemia in normal and diabetic dogs. Journal of Small Animal Practice, 31, 229–233.

[jsap70022-bib-0006] Breyton, A.E. , Goux, A. , Lambert‐Porcheron, S. , Meynier, A. , Sothier, M. , VanDenBerghe, L. et al. (2020) Starch digestibility modulation significantly improves glycaemic variability in type 2 diabetic subjects: a pilot study. Nutrition, Metabolism, and Cardiovascular Diseases, 31, 237–246. 10.1016/j.numecd.2020.08.010 32988721

[jsap70022-bib-0007] Briens, J.M. , Subramaniam, M. , Kilgour, A. , Loewen, M.E. , Desai, K.M. , Adolphe, J.L. et al. (2021) Glycemic, insulinemic and methylglyoxal postprandial responses to starches alone or in whole diets in dogs versus cats: relating the concept of glycemic index to metabolic responses and gene expression. Comparative Biochemistry and Physiology, Part A: Molecular & Integrative Physiology, 257, 110973. 10.1016/j.cbpa.2021.110973 33933629

[jsap70022-bib-0008] Camps, S.G. , Kaur, B. , Lim, J. , Loo, Y.T. , Pang, E. , Ng, T. et al. (2021) Improved glycaemic control and variability: application of healthy ingredients in Asian Staples. Nutrients, 13, 3102. 34578981 10.3390/nu13093102 PMC8468310

[jsap70022-bib-0009] Ceriello, A. , Monnier, L. & Owens, D. (2019) Glycaemic variability in diabetes: clinical and therapeutic implications. Lancet Diabetes and Endocrinology, 7, 221–230. 30115599 10.1016/S2213-8587(18)30136-0

[jsap70022-bib-0010] Chrzanowski, J. , Grabia, S. , Michalak, A. , Wielgus, A. , Wykrota, J. , Mianowska, B. et al. (2023) GlyCulator 3.0: a fast, easy‐to‐use analytical tool for CGM data analysis, aggregation, centre benchmarking, and data sharing. Diabetes Care, 46, e3–e5. 36356162 10.2337/dc22-0534 PMC9918444

[jsap70022-bib-0011] Corradini, S. , Pilosio, B. , Dondi, F. , Linari, G. , Testa, S. , Brugnoli, F. et al. (2016) Accuracy of flash glucose monitoring system in diabetic dogs. Journal of Veterinary Internal Medicine, 30, 983–988. 27318663 10.1111/jvim.14355 PMC5094557

[jsap70022-bib-0012] Corsato, A.I. , Keller, L.C. , Waldy, C. & Aldrich, C.G. (2021) Extrusion processing modifications of a dog kibble at large scale alter levels of starch available to animal enzymatic digestion. Food, 10, 2526. 10.3390/foods10112526 PMC8621379 34828807

[jsap70022-bib-0013] Del Baldo, F. , Canton, C. , Testa, S. , Swales, H. , Drudi, I. , Golinelli, S. et al. (2020) Comparison between a flash glucose monitoring system and a portable blood glucose meter for monitoring dogs with diabetes mellitus. Journal of Veterinary Internal Medicine, 34, 2296–2305. 33124730 10.1111/jvim.15930 PMC7694810

[jsap70022-bib-0014] Del Baldo, F. & Fracassi, F. (2023) Continuous glucose monitoring in dogs and cats: application of new technology to an old problem. Veterinary Clinics of North America Small Animal Practice, 53, 591–613. 36854635 10.1016/j.cvsm.2023.01.008

[jsap70022-bib-0015] Del Baldo, F. , Magna, L. , Dondi, F. , Maramieri, P. , Catrina, O.M. , Corradini, S. et al. (2020) Comparison of serum fructosamine and glycated haemoglobin values for assessment of glycaemic control in dogs with diabetes mellitus. American Journal of Veterinary Research, 81, 233–242. 32101039 10.2460/ajvr.81.3.233

[jsap70022-bib-0016] Elliott, K.F. , Rand, J.S. , Fleeman, L.M. , Morton, J.M. , Litster, A.L. , Biourge, V.C. et al. (2012) A diet lower in digestible carbohydrate results in lower postprandial glucose concentrations compared with a traditional canine diabetes diet and an adult maintenance diet in healthy dogs. Research in Veterinary Science, 93, 288–295. 21944832 10.1016/j.rvsc.2011.07.032

[jsap70022-bib-0017] European Society of Veterinary Endocrinology . (2020) Project ALIVE. Available from: https://www.esve.org/alive/intro.aspx [Accessed 16th June 2025].

[jsap70022-bib-0018] Fleeman, L.M. , Rand, J.S. & Markwell, P.J. (2009) Lack of advantage of high‐fibre, moderate‐carbohydrate diets in dogs with stabilised diabetes. Journal of Small Animal Practice, 50, 604–614. 19814767 10.1111/j.1748-5827.2009.00817.x

[jsap70022-bib-0019] Gibson, M.W. & Alavi, S. (2013) Pet food processing: understanding transformations in starch during extrusion and baking. Cereal Foods World, 58, 232–236.

[jsap70022-bib-0020] Graham, P.A. , Maskell, I.E. , Rawlings, J.M. , Nash, A.S. & Markwell, P.J. (2002) Influence of a high fibre diet on glycaemic control and quality of life in dogs with diabetes mellitus. Journal of Small Animal Practice, 43, 67–73. 11873951 10.1111/j.1748-5827.2002.tb00031.x

[jsap70022-bib-0021] Hewson‐Hughes, A.K. , Gilham, M.S. , Upton, S. , Colyer, A. , Butterwick, R. & Miller, A.T. (2011) The effect of dietary starch level on postprandial glucose and insulin concentrations in cats and dogs. British Journal of Nutrition, 106, S105–S109. 22005401 10.1017/S0007114511001887

[jsap70022-bib-0022] Jung, H.S. (2015) Clinical implications of glucose variability: chronic complications of diabetes. Endocrinology and Metabolism, 30, 167–174. 26194076 10.3803/EnM.2015.30.2.167 PMC4508260

[jsap70022-bib-0023] Kimmel, S.E. , Michel, K. , Hess, R.S. & Ward, C.R. (2000) Effects of insoluble and soluble dietary fibre on glycaemic control in dogs with naturally occurring insulin‐dependent diabetes mellitus. Journal of the American Veterinary Medical Association, 216, 1076–1081. 10754666 10.2460/javma.2000.216.1076

[jsap70022-bib-0024] Krämer, A.L. , Riederer, A. , Fracassi, F. , Boretti, F.S. , Sieber‐Ruckstuhl, N.S. , Lutz, T.A. et al. (2020) Glycaemic variability in newly diagnosed diabetic cats treated with the glucagon‐like peptide‐1 analogue exenatide extended release. Journal of Veterinary Internal Medicine, 34, 2287–2295. 33001499 10.1111/jvim.15915 PMC7694851

[jsap70022-bib-0025] Linari, G. , Fleeman, L. , Gilor, C. , Giacomelli, L. & Fracassi, F. (2022) Insulin glargine 300 U/mL for the treatment of feline diabetes mellitus. Journal of Feline Medicine and Surgery, 24, 168–176. 34009061 10.1177/1098612X211013018 PMC10812176

[jsap70022-bib-0026] Miller, M. , Pires, J. , Crakes, K. , Greathouse, R. , Quach, N. & Gilor, C. (2021) Day‐to‐day variability of porcine lente, insulin glargine 300 U/mL and insulin degludec in diabetic dogs. Journal of Veterinary Internal Medicine, 35, 2131–2139. 34241910 10.1111/jvim.16178 PMC8478047

[jsap70022-bib-0027] Nelson, R.W. (2015) Canine diabetes mellitus. In: Nelson, R.W. , Reusch, C.E. , Scott‐Moncrieff, J.C. , Feldman, E.C. & Behrend, E.N. (Eds.) Canine and feline endocrinology, 4th edition. St Louis, MO: Elsevier Saunders, pp. 213–257.

[jsap70022-bib-0028] Nelson, R.W. , Duesberg, C.A. , Ford, S.L. , Feldman, E.C. , Davenport, D.J. , Kiernan, C. et al. (1998) Effect of dietary insoluble fibre on control of glycaemia in dogs with naturally acquired diabetes mellitus. Journal of the American Veterinary Medical Association, 212, 380–386. 9470048

[jsap70022-bib-0029] Oliveira, M.C. , Brunetto, M.A. , da Silva, F.L. , Jeremias, J.T. , Tortola, L. , Gomes, M.O. et al. (2014) Evaluation of the owner’s perception in the use of homemade diets for the nutritional management of dogs. Journal of Nutritional Science, 25, e23. 10.1017/jns.2014.24 PMC4473168 26101592

[jsap70022-bib-0030] Parker, V.J. & Hill, R.C. (2023) Nutritional management of cats and dogs with diabetes mellitus. Veterinary Clinics of North America: Small Animal Practice, 53, 657–674. 36858905 10.1016/j.cvsm.2023.01.007

[jsap70022-bib-0031] Rasmussen, L. , Christensen, M.L. , Poulsen, C.W. , Rud, C. , Christensen, A.S. , Andersen, J.R. et al. (2020) Effect of high versus low carbohydrate intake in the morning on glycaemic variability and glycaemic control measured by continuous blood glucose monitoring in women with gestational diabetes mellitus – a randomised crossover study. Nutrients, 12, 475. 32069857 10.3390/nu12020475 PMC7071236

[jsap70022-bib-0032] Remillard, R.L. (2008) Homemade diets: attributes, pitfalls, and a call for action. Topics in Companion Animal Medicine, 23, 137–142. 18656841 10.1053/j.tcam.2008.04.006

[jsap70022-bib-0033] Shea, E.K. & Hess, R.S. (2021) Assessment of postprandial hyperglycemia and circadian fluctuation of glucose concentrations in diabetic dogs using a flash glucose monitoring system. Journal of Veterinary Internal Medicine, 35, 843–852. 33522022 10.1111/jvim.16046 PMC7995415

[jsap70022-bib-0034] Sima, P. , Vannucci, L. & Vetvicka, V. (2018) β‐Glucans and cholesterol. International Journal of Molecular Medicine, 41, 1799–1808. 29393350 10.3892/ijmm.2018.3411 PMC5810204

[jsap70022-bib-0035] Tardo, A.M. , Fleeman, L.M. , Fracassi, F. , Berg, A.S. , Guarino, A.L. & Gilor, C. (2024) A dose titration protocol for once‐daily insulin glargine 300 U/mL for the treatment of diabetes mellitus in dogs. Journal of Veterinary Internal Medicine, 38, 2120–2128. 38831362 10.1111/jvim.17106 PMC11256126

[jsap70022-bib-0036] Tay, J. , Thompson, C.H. & Brinkworth, G.D. (2015) Glycaemic variability: assessing glycemia differently and the implications for dietary management of diabetes. Annual Review of Nutrition, 35, 389–424. 10.1146/annurev-nutr-121214-104422 25974701

[jsap70022-bib-0037] Teixeira, F.A. & Brunetto, A. (2017) Nutritional factors related to glucose and lipid modulation in diabetic dogs: literature review. Brazilian Journal of Veterinary Research and Animal Science, 54, 330–341.

[jsap70022-bib-0038] Teixeira, F.A. , Machado, D.P. , Jeremias, J.T. , Queiroz, M.R. , Pontieri, C.F.F. & Brunetto, M.A. (2020) Starch sources influence lipidaemia of diabetic dogs. BMC Veterinary Research, 16, 2. 31900155 10.1186/s12917-019-2224-y PMC6942337

[jsap70022-bib-0039] Teshima, E. , Brunetto, M.A. , Teixeira, F.A. , Gomes, M.O.S. , Lucas, S.R.R. , Pereira, G.T. et al. (2021) Influence of type of starch and feeding management on glycaemic control in diabetic dogs. Journal of Animal Physiology and Animal Nutrition, 105, 1192–1202. 33904623 10.1111/jpn.13556

[jsap70022-bib-0040] Tettamanzi, F. , Bagnardi, V. , Louca, P. , Nogal, A. , Monti, G.S. , Mambrini, S.P. et al. (2021) A high protein diet is more effective in improving insulin resistance and glycaemic variability compared to a mediterranean diet – a cross‐over controlled inpatient dietary study. Nutrient, 13, 4380. 10.3390/nu13124380 PMC8707429 34959931

[jsap70022-bib-0041] Truswell, A.S. (1992) Glycaemic index of foods. European Journal of Clinical Nutrition, 46, S91–S101. 1330533

[jsap70022-bib-0042] Vergès, B. , Pignol, E. , Rouland, A. , Bouillet, B. , Baillot‐Rudoni, S. , Quilot, E. et al. (2022) Glycemic variability assessment with a 14‐day continuous glucose monitoring system: when and how long to measure MAGE (mean amplitude of glucose excursion) for optimal reliability? Journal of Diabetes Science and Technology, 16, 982–987. 33567877 10.1177/1932296821992060 PMC9264451

[jsap70022-bib-0043] Zeugswetter, F.K. & Sellner, A. (2020) Flash glucose monitoring in diabetic dogs: a feasible method for evaluating glycemic control. Tierarztliche Praxis Ausgabe K Kleintiere Heimtiere, 48, 330–338. 33086409 10.1055/a-1239-4739

[jsap70022-bib-0044] Zini, E. , Salesov, E. , Dupont, P. , Moretto, L. , Contiero, B. , Lutz, T.A. et al. (2018) Glucose concentrations after insulin‐induced hypoglycemia and glycaemic variability in healthy and diabetic cats. Journal of Veterinary Internal Medicine, 32, 978–985. 29603806 10.1111/jvim.15134 PMC5980264

