# Supplementary material for: Effect of a homemade diet compared to a commercial diet on glycaemic variability and glycaemic control assessed by continuous glucose monitoring system in diabetic dogs: a randomised crossover study
Source: J Small Anim Pract. 2025 Aug 22;67(2):172–80. doi: 10.1111/jsap.70022 (PMC12883309; doi:10.1111/jsap.70022)
Supplement: Supplementary file 1 — Data S1. [file JSAP-67-172-s001.docx]

Supplementary Material 1: Agreeing Language in Veterinary Endocrinology (ALIVE) Diabetic Clinical Score of the European Society of Veterinary Endocrinology (ESVE). Range total score: 0-12. The treatment aim is to have lowest score possible without unacceptably high risk of hypoglycaemia. From: European Society of Veterinary Endocrinology. Project ALIVE (2020) https://www. esve.org/alive/intro.aspx.

| **Factor** | **Score** |
| --- | --- |
| **Unintended Weight Loss**  0 = None, or gained since last examined  1 = Mild (<5% loss)  2 = Moderate (5-10% loss)  3 = Severe (>10% loss) | **…** |
| **Polyuria and polydipsia**  0 = Normal  1 = Mild (some increase noted by owner)  2 = Moderate (increased filling of water bowl)  3 = Severe (constantly at bowl) | **…** |
| **Appetite**  0 = Normal or decreased appetite (if decreased appetite exclude DKA or concurrent disease)  1 = Mild polyphagia (finishes eagerly)  2 = Moderate polyphagia (finished eagerly and begs for more)  3 = Severe polyphagia (obsessed with food) | **…** |
| **Attitude/activity**  0 = Normal  1 = Mild decrease (a bit less running and jumping)  2 = Moderate decrease (a lot less running and jumping)  3 = Severe decrease (lying all the time) (*consider DKA in the ill patient with diabetes mellitus) | **…** |
| **Total score=** | **…** |
